# Functional categorization of de novo transcriptome assembly of *Vanilla planifolia* Jacks. potentially points to a translational regulation during early stages of infection by *Fusarium oxysporum* f. sp. *vanillae*

**DOI:** 10.1186/s12864-019-6229-5

**Published:** 2019-11-08

**Authors:** Marco Tulio Solano-De la Cruz, Jacel Adame-García, Josefat Gregorio-Jorge, Verónica Jiménez-Jacinto, Leticia Vega-Alvarado, Lourdes Georgina Iglesias-Andreu, Esteban Elías Escobar-Hernández, Mauricio Luna-Rodríguez

**Affiliations:** 1 Instituto de Biotecnología y Ecología Aplicada (INBIOTECA), Universidad Veracruzana, Avenida de las Culturas Veracruzanas s/n, Xalapa, Veracruz, Mexico; 2 Instituto de Ecología, Universidad Nacional Autónoma de México, Circuito Exterior S/N anexo, Jardín Botánico exterior, Ciudad Universitaria, Ciudad de México, Mexico; 3 Tecnológico Nacional de México, Instituto Tecnológico de Úrsulo Galván, Úrsulo Galván, Veracruz, Mexico; 4 Consejo Nacional de Ciencia y Tecnología - Centro de Investigación en Biotecnología Aplicada, Instituto Politécnico Nacional (CIBA-IPN), Av. Insurgentes Sur 1582, Col. Crédito Constructor, Del. Benito Juárez, 03940 Ciudad de México, Mexico; 5 Unidad Universitaria de Secuenciación Masiva y Bioinformática, Instituto de Biotecnología, Universidad Nacional Autónoma de México, Cuernavaca, Morelos Mexico; 6 Instituto de Ciencias Aplicadas y Tecnología, Universidad Nacional Autónoma de México, Ciudad de México, Mexico; 7 Unidad de Genómica Avanzada, Langebio, Cinvestav, Km 9.6 Libramiento Norte Carretera León, Irapuato, Guanajuato, Mexico; 8 Laboratorio de Genética e Interacciones Planta Microorganismos, Facultad de Ciencias Agrícolas, Universidad Veracruzana. Circuito Gonzalo Aguirre Beltrán s/n, Zona Universitaria, Xalapa, Veracruz Mexico

**Keywords:** Translational regulation, Biological defense, Transcriptional reprogramming, Biotic stress, Ribosomal proteins

## Abstract

**Background:**

Upon exposure to unfavorable environmental conditions, plants need to respond quickly to maintain their homeostasis. For instance, physiological, biochemical and transcriptional changes occur during plant-pathogen interaction. In the case of *Vanilla planifolia* Jacks., a worldwide economically important crop, it is susceptible to *Fusarium oxysporum* f. sp. *vanillae* (*Fov*). This pathogen causes root and stem rot (RSR) in vanilla plants that lead to plant death. To investigate how vanilla plants, respond at the transcriptional level upon infection with *Fov*, here we employed the RNA-Seq approach to analyze the dynamics of whole-transcriptome changes during two-time frames of the infection.

**Results:**

Analysis of global gene expression profiles upon infection by *Fov* indicated that the major transcriptional change occurred at 2 days post-inoculation (dpi), in comparison to 10 dpi. Briefly, the RNA-Seq analysis carried out in roots found that 3420 and 839 differentially expressed genes (DEGs) were detected at 2 and 10 dpi, respectively, as compared to the control. In the case of DEGs at 2 dpi, 1563 genes were found to be up-regulated, whereas 1857 genes were down-regulated. Moreover, functional categorization of DEGs at 2 dpi indicated that up-regulated genes are mainly associated to translation, whereas down-regulated genes are involved in cell wall remodeling. Among the translational-related transcripts, ribosomal proteins (RPs) were found increased their expression exclusively at 2 dpi.

**Conclusions:**

The screening of transcriptional changes of *V. planifolia* Jacks upon infection by *F*ov provides insights into the plant molecular response, particularly at early stages of infection. The accumulation of translational-related transcripts at early stages of infection potentially points to a transcriptional reprogramming coupled with a translational regulation in vanilla plants upon infection by *Fov*. Altogether, the results presented here highlight potential molecular players that might be further studied to improve *Fov*-induced resistance in vanilla plants.

## Background

Throughout evolution, plants have developed multiple defense strategies to cope with pathogens. The first defense line consists of pre-existing physical and chemical barriers, which restrict their entry [1]. In addition to these constitutive barriers, plants have developed an immune response mechanism that is based on the detection of elicitor compounds derived from pathogens, known as Pathogen-Associated Molecular Patterns (PAMPs) [2]. Such defense response activated by the PAMPs or PAMP-Triggered Immunity (PTI), usually restricts the proliferation of the pathogen [3–7]. However, some pathogens have circumvented this response by developing effector proteins that interfere or suppress PTI [8–10]. In this sense, the so-called co-evolutionary ‘arms race’ between plants and pathogens has defined the establishment of the Effector-Triggered Immunity (ETI), a defense line that begins with the recognition of PAMPs by plant pattern recognition receptors (PRRs) [11]. The signals generated by PRRs are transduced through Mitogen-activated Protein Kinases (MAPKs), which in turn activate transcription factors for gene regulation that leads to a proper plant defense response [12]. Among the plant responses, the Hypersensitive Response (HR), the programmed cell death, the expression of proteins related to pathogenesis or the lignification of the cell wall are included [13–18].

*Vanilla planifolia* Jacks. is one of the most economically relevant orchids. It is produced extensively in several countries and is the main natural source of one of the most widely used flavoring agents in the world, vanillin [19, 20]. Its cultivation has spread throughout the world, with Madagascar and Indonesia as the leaders of annual production (35.5 and 34.5%, respectively), followed by China (13.7%) and Papua New Guinea (4.1%) [21–25]. Although Mexico is the center of domestication and diversification of this crop, vanillin production is positioned in the fifth place, contributing to only 4.0% of world production [20]. Importantly, vanilla plants are susceptible to parasites and pathogens. The most lethal pathogen that afflicts vanilla is *Fov*, a pathogenic form of the genus *Fusarium* that specifically infects this plant species [22, 25, 26]. This pathogen causes RSR, as well as the colonization of vascular tissues that finally leads to plant death. Several studies indicate that *V. planifolia* has a high susceptibility and incidence of *Fov* [25, 27, 28]. For instance, infection of vanilla plants by this pathogen is capable of destroying 65% of the plantation [22, 25, 26]. The lack of genetic variability of *V. planifolia* is another factor that worsens the scenario [26, 29, 30]. Thus, given the economic importance of *V. planifolia*, is mandatory to do an effort to elucidate the overall plant response upon infection by this pathogen, likewise, has been done in other crops [31–33]. Moreover, since inferences from mRNA expression data are valuable as it reflects changes with a biological meaning, we looked into the transcriptome of *V. planifolia* roots exposed to *Fov*, to figure out the responsive mechanisms at early (2 days after inoculation, 2dpi) and later (10 days after inoculation, 10 dpi) stages of infection. Gene expression profiles indicated that major transcriptional changes occur at 2 dpi. Accordingly, vanilla plants accumulate transcripts associated to several processes, but mostly translational regulation-related transcripts. Thus, this study provides the identification of molecular players in plant-pathogen interaction between *V. planifolia* and *F. oxysporum* f. sp. *vanillae*, particularly a transcriptional reprogramming coupled with a translational regulation. Our study is aimed to understand the response of vanilla plants, which could help to fight the most damaging disease of vanilla caused by *Fov*.

## Results

### Assembly of the transcriptome of *V. planifolia* roots exposed to *Fov*

**Fig. 1:**
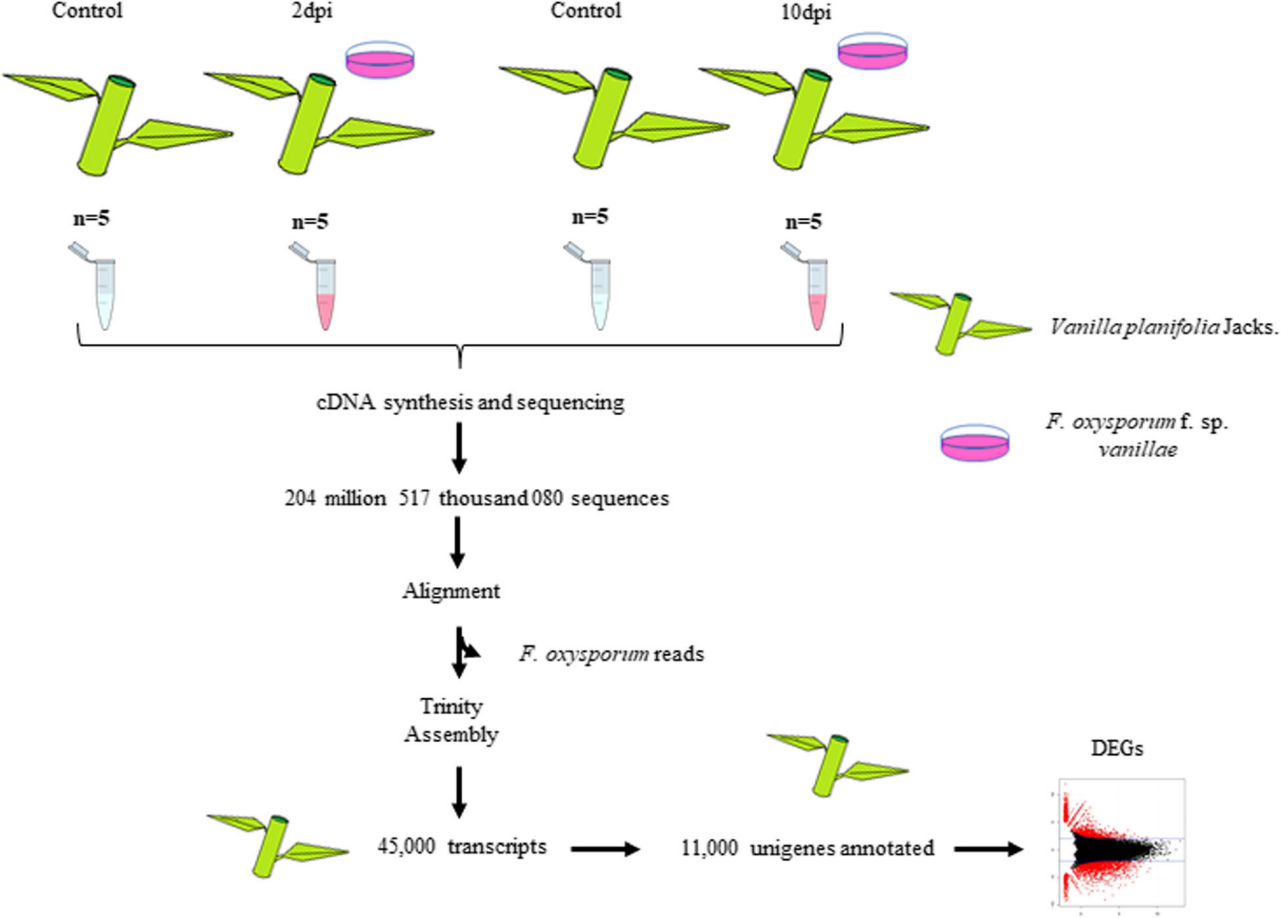
Flow diagram outlining the experimental design and key steps in the process of the de novo transcriptome assembly for *V. planifolia* plants upon infection by *Fov*. Total RNA from non-treated (Control; C) and treated (2 and 10 dpi) plants were converted to cDNA and subjected to high- throughput sequencing. For details, see Materials and Methods

The transcriptome of vanilla roots exposed to *Fov* was assessed with Illumina sequencing at 2 and 10 dpi. A total of 12 cDNA libraries were paired-end sequenced using the NextSeq 500 system. Sequencing data of these libraries were obtained corresponding to three biological replicates (control and treatment), covering two frames of time along the infection process (Fig. 1). In brief, six libraries corresponding to control and treatment at 2 dpi, as well as six libraries at 10 dpi, produced more than 204 million reads (Fig. 1). Such data were submitted to the GEO platform of NCBI-GenBank (Accession number: GSE134155). To analyze and compare the dispersion of the treatments with respect to the control samples, PCA analyzes were carried out (Additional file 1: Figure S1; and Additional file 2: Figure S2). Additional file 1: Figure S1 corresponds to the treatment and control at 2 dpi, whereas Additional file 2: Figure S2 corresponds to the treatment and control at 10 dpi; respectively. Pre-processing of raw sequencing reads was carried out with FastQC, which indicated a good per base quality. The results of the quality analysis applied to the raw data, with FastQC software, are hosted under the following link: http://www.uusmb.unam.mx/reportes/170308/Project_MTulio.html (Additional file 3: Table S1). Filtering reads that correspond to the pathogen used at 2 and 10 dpi, discarded 5.33 and 39%, respectively. On the other hand, even that control plants were not inoculated with the fungus, 5% (2 dpi) and 6.48% (10 dpi) of reads aligned to the genome of *F. oxysporum* f. sp. *lycopersici*, excluding such reads for subsequent analyzes (Fig. 1). The de novo transcriptome assembly of vanilla resulted in about 45,000 transcripts (Additional file 4: Figure S3). The statistics of the transcriptome assembly carried out by TransRate v1.0.3 [34] can be found in (Additional file 5: Table S2) (Accession number: GSE134155). In addition, the results of processing data, which involved sequencing statistics of raw data and filtered data, statistics of the sequence alignments vs. the de novo transcriptome assembly [35], as well as non-aligned sequences and records of sequences that were cleaned are presented in (Additional file 3: Table S1). The generated transcripts were mapped against the plant databases, using the BUSCO software, obtaining about 99% of complete orthologous. Additional file 1: Figure S1 shows the results of the annotation of the vanilla transcriptome with Blast2GO, finding about 11,000 unigenes out of the total 45,000 assembled transcripts (Additional file 4: Figure S3). Among the main functional categories of gene ontology obtained were plant development, plant growth, cell proliferation, signaling, response to stimuli and response to stress. Moreover, counting of reads on the assembled transcripts resulted in approximately 30% of transcripts that fulfilled the counts per million required for the subsequent identification of DEGs. Altogether, the assessment of the transcriptome of *V. planifolia* roots exposed to *Fov* revealed that several plant and cellular processes are impacted during the two frames of time evaluated.

### Analysis of gene expression and functional categorization of DEGs at 2 and 10 dpi

**Fig. 2:**
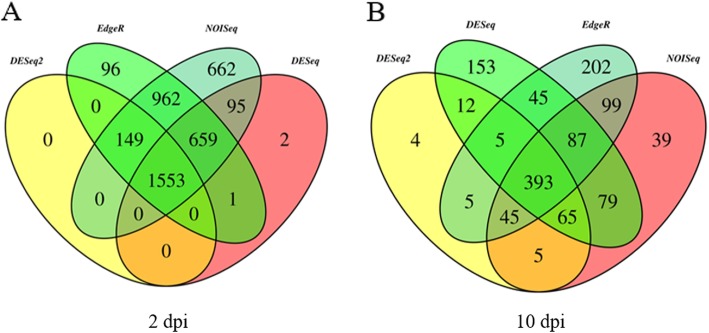
Venn diagrams showing the degree of overlap between DEGs obtained with different methods. **a** Number of DEGs obtained by DESeq, DESeq2, NOISeq and EdgeR for data set at 2 dpi. **b** Number of DEGs obtained for data set at 10 dpi with the same methods as shown in **a**. Results from each method are shown with different colors

**Fig. 3:**
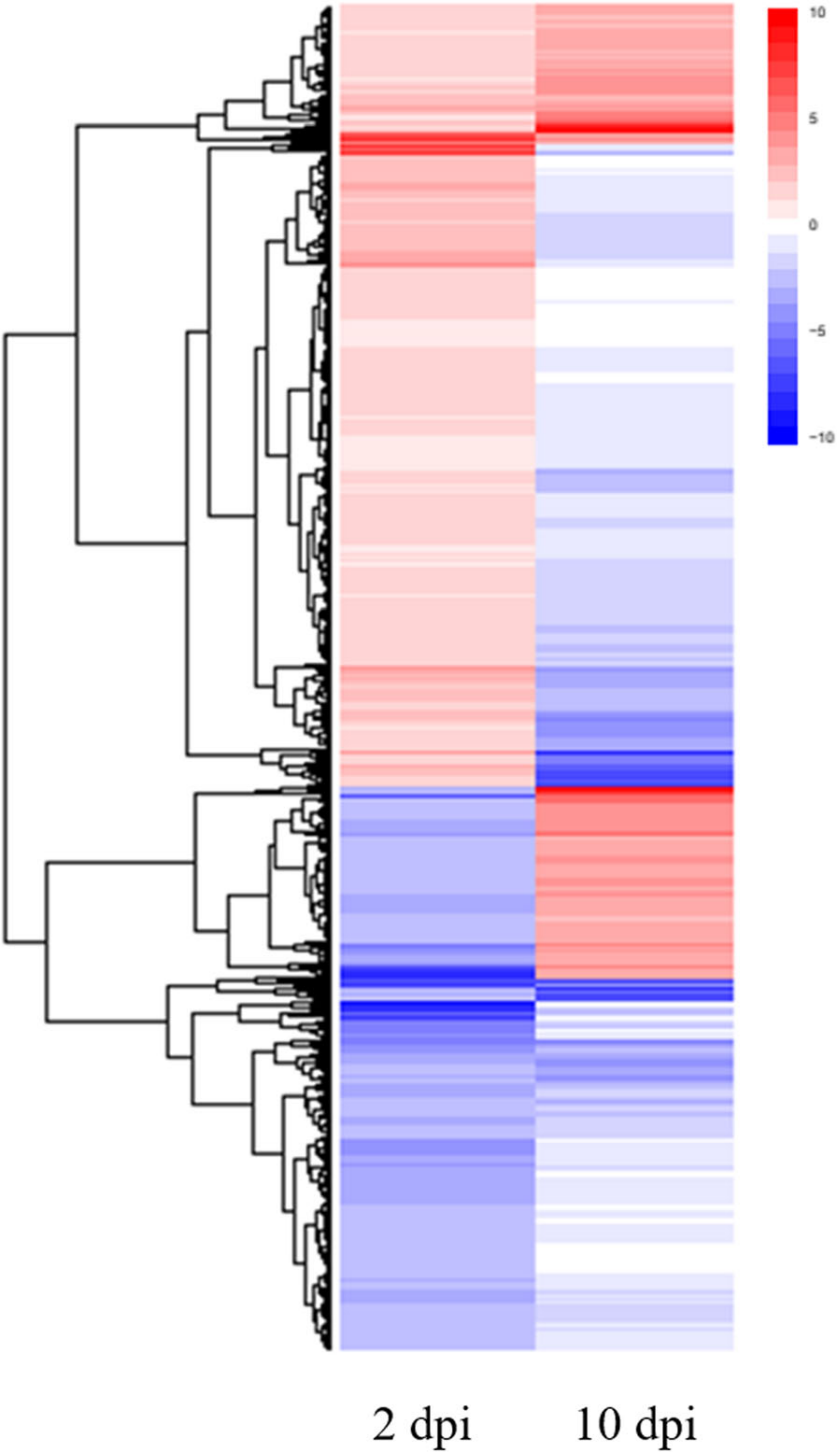
Overall expression patterns of DEGs at 2 and 10 dpi. Heat maps of data sets at 2 (3420 DEGs) and 10 dpi (881 DEGs) are shown. The heat maps were made using the ggplot2 included in R package [36]

For the identification of unigenes with changes in expression levels at 2 and 10 dpi, differential gene expression analysis was carried out using several approaches such as DESeq, DESeq2, NOISeq and EdgeR. For libraries corresponding to 2 dpi, 2310, 1702, 4080 and 3420 DEGs were obtained with DESeq, DESeq2, NOISeq and EdgeR, respectively (Fig. 2) (Additional file 6: Table S3). On the other hand, analysis of DEGs at 10 dpi revealed that 812, 534, 839 and 881 DEGs were obtained with DESeq, DESeq2, NOISeq and EdgeR, respectively (Fig. 2) (Additional file 6: Table S3). As EdgeR is the most popular method and taking into account that this method included the vast majority of DEGs, EdgeR was selected for the subsequent analysis (Fig. 2). In that sense, two lists were obtained, one corresponding to the treatment at 2 dpi containing 3420 DEGs and the other corresponding to the treatment at 10 dpi with 881 DEGs. In the case of DEGs at 2 dpi, 1563 genes were found to be up-regulated, whereas 1857 genes were down-regulated. On the other hand, classification of DEGs at 10 dpi as up- and down-regulated genes, resulted in 250 and 631 genes, respectively. An overview of the transcriptional change at 2 and 10 dpi is shown in Fig. 3. At a glance, subsets of certain DEGs showed contrasting expression profiles if both treatments are compared (Fig. 3).

**Fig. 4:**
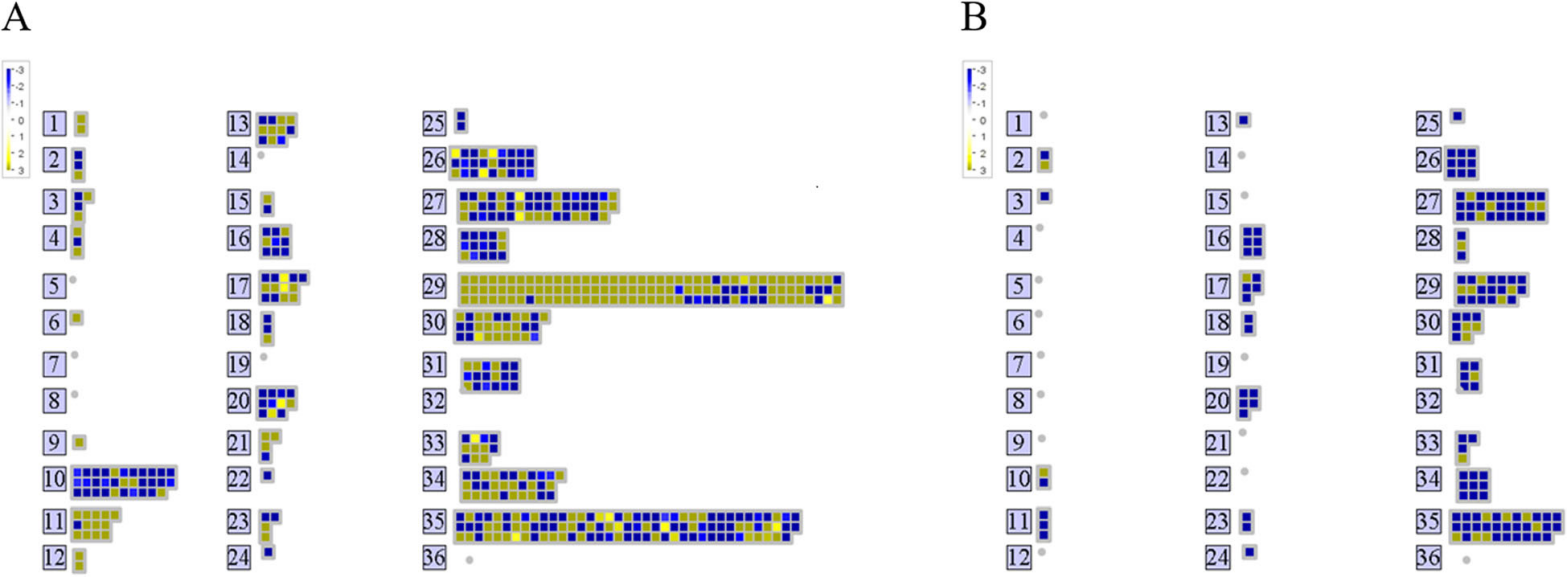
MapMan analysis of DEGs showing their expression profiles at 2 and 10 dpi. **a** Heat map of DEGs at 2 dpi. **b** Heat map of DEGs at 10 dpi. The numbers correspond to different MapMan functional categories of gene ontology as described below: 1 PS, 2, major CHO metabolism, 3 minor CHO metabolism, 4 glycolysis, 6 gluconeogenese/glyoxylate cycle, 9 mitochondrial electron transport/ATP synthesis, 10 cell wall, 11 lipid metabolism, 12 N-metabolism, 13 amino acid metabolism, 15 metal handling, 16 secondary metabolism, 17 hormone metabolism, 18 Co-factor and vitamin metabolism, 20 stress, 21 redox, 22 polyamine metabolism, 23 nucleotide metabolism, 24 Biodegradation of Xenobiotics, 25 C1-metabolism, 26 misc., 27 RNA, 28 DNA, 29 protein, 30 signaling, 31 cell, 33 development, 34 transport, 35 not assigned

The lack of reference genome for *V. planifolia* forced to check orthology with available genomes for which annotation is complete. Accordingly, orthologs of Arabidopsis corresponding to DEGs at 2 and 10 dpi were obtained, resulting in 603 and 278 orthologs, respectively (Additional file 7: Table S4). As a first approach to elucidate the putative functions of DEGs at 2 and 10 dpi, gene orthologs were submitted to MapMan [37]. Pathway analysis of DEGs with *P*-value cut-off of ≤0.05 was carried out on Arabidopsis pathway genes. Accordingly, 603 (2 dpi) and 278 (10 dpi) DEGs were analyzed with MapMan, from which only 535 and 149 were categorized, respectively (Fig. 4). Visualization of the DEGs assigned to functional categories revealed that orthologs with differential expression at 2 dpi showed most enriched categories than that of 10 dpi (Fig. 4). Remarkably, most of data points contained within the functional categories at 2 dpi were up-regulated genes, whereas down-regulated genes were mostly associated to functional categories at 10 dpi (Fig. 4). Among the most enriched categories, genes encoding products involved in regulation of transcription (27) and protein synthesis (29) were observed in both cases (2 and 10 dpi) (Fig. 4). However, the number of genes associated to those functional categories was contrasting. For instance, whereas only 20 data points were found within the category of protein for DEGs at 10 dpi, 123 were found in the case of data corresponding to 2 dpi (Fig. 4). Other enriched categories at 2 dpi were cell wall (10), lipid metabolism (11), amino acid metabolism (13), secondary metabolism (16) and hormone metabolism (17) (Fig. 4). Thus, the functional categorization of DEGs suggest that the major transcriptional change occurs at early stages of infection, namely at 2 dpi.

To further comprehend the functions of DEGs at 2 and 10 dpi, an analysis according to the enrichment of GO terms was carried out using the orthologs. Such analysis in the platform of agriGO resulted in the main functional categories associated to DEGs at 2 dpi (Table 1), but not for DEGs at 10 dpi. For up-regulated genes at 2 dpi, 18, 4 and 30 categories were significantly enriched, corresponding to biological process (P), molecular function (F) and cellular component (C), respectively (Table 1). The GO Term Enrichment Analysis was also performed with the PANTHER classification system software (v.14.0), to determine the categories of Gene Ontology significantly enriched in DEGs at 2 dpi, present in the up and down-regulated transcripts (Additional file 8: Table S5, and Additional file 9: Table S6 respectively). In the case of down-regulated genes, 9, 3 and 3 categories were found significantly enriches for P, F and C, respectively (Table 1). A schematic representation of biological processes shown in Table 1 allowed to appreciate that translation and cell wall modification were the main processes overrepresented in up- and down-regulated genes, respectively (Additional file 10: Figure S4). Taken together, the functional categorization of DEGs not only suggests that the major transcriptional change occurs at early stages of infection (2 dpi), but also indicates that up-regulated genes are mainly associated to translation, whereas down-regulated genes are involved in cell wall remodeling.

**Table 1:** Gene Ontology categories significantly enriched in DEGs at 2 dpi in the infection caused by *Fusarium* in vanilla

DEGs	GO term	Ontology	Description	*p*-value	FDR
Up-Regulated	GO:0006412	P	translation	2.90E-41	2.10E-38
	GO:0034645	P	cellular macromolecule biosynthetic process	6.50E-29	1.90E-26
	GO:0009058	P	biosynthetic process	7.90E-29	1.90E-26
	GO:0009059	P	macromolecule biosynthetic process	1.00E-28	1.90E-26
	GO:0044249	P	cellular biosynthetic process	2.80E-28	4.00E-26
	GO:0019538	P	protein metabolic process	1.10E-26	1.30E-24
	GO:0010467	P	gene expression	1.80E-24	1.90E-22
	GO:0044267	P	cellular protein metabolic process	2.70E-24	2.40E-22
	GO:0044238	P	primary metabolic process	4.00E-24	3.20E-22
	GO:0043170	P	macromolecule metabolic process	6.60E-21	4.70E-19
	GO:0008152	P	metabolic process	1.40E-20	9.10E-19
	GO:0044260	P	cellular macromolecule metabolic process	4.40E-19	2.60E-17
	GO:0044237	P	cellular metabolic process	3.40E-17	1.90E-15
	GO:0009987	P	cellular process	1.60E-16	8.30E-15
	GO:0042254	P	ribosome biogenesis	2.70E-16	1.30E-14
	GO:0022613	P	ribonucleoprotein complex biogenesis	6.60E-16	3.00E-14
	GO:0044085	P	cellular component biogenesis	4.40E-11	1.80E-09
	GO:0009791	P	post-embryonic development	3.90E-06	0.00016
	GO:0003735	F	structural constituent of ribosome	4.20E-57	1.40E-54
	GO:0005198	F	structural molecule activity	5.20E-50	8.30E-48
	GO:0008135	F	translation factor activity, nucleic acid binding	1.20E-07	1.30E-05
	GO:0003746	F	translation elongation factor activity	6.10E-07	4.90E-05
	GO:0022626	C	cytosolic ribosome	4.60E-65	1.00E-62
	GO:0044445	C	cytosolic part	1.00E-57	1.10E-55
	GO:0033279	C	ribosomal subunit	5.50E-56	4.10E-54
	GO:0005840	C	ribosome	1.20E-55	6.50E-54
	GO:0030529	C	ribonucleoprotein complex	2.00E-48	8.80E-47
	GO:0043232	C	intracellular non-membrane-bounded organelle	3.70E-45	1.20E-43
	GO:0043228	C	non-membrane-bounded organelle	3.70E-45	1.20E-43
	GO:0005829	C	cytosol	3.90E-43	1.10E-41
	GO:0022625	C	cytosolic large ribosomal subunit	3.20E-40	7.70E-39
	GO:0015934	C	large ribosomal subunit	1.10E-35	2.40E-34
	GO:0044422	C	organelle part	3.30E-30	6.00E-29
	GO:0044446	C	intracellular organelle part	3.20E-30	6.00E-29
	GO:0032991	C	macromolecular complex	7.00E-27	1.20E-25
	GO:0022627	C	cytosolic small ribosomal subunit	1.20E-22	1.80E-21
	GO:0015935	C	small ribosomal subunit	1.10E-20	1.60E-19
	GO:0044444	C	cytoplasmic part	2.30E-19	3.20E-18
	GO:0005737	C	cytoplasm	2.80E-18	3.70E-17
	GO:0005730	C	nucleolus	2.70E-16	3.20E-15
	GO:0043229	C	intracellular organelle	4.40E-14	4.90E-13
	GO:0005622	C	intracellular	4.40E-14	4.90E-13
	GO:0043226	C	organelle	4.70E-14	4.90E-13
	GO:0031981	C	nuclear lumen	1.00E-13	1.00E-12
	GO:0044424	C	intracellular part	2.70E-13	2.60E-12
	GO:0043233	C	organelle lumen	2.20E-12	1.90E-11
	GO:0070013	C	intracellular organelle lumen	2.20E-12	1.90E-11
	GO:0044428	C	nuclear part	2.50E-12	2.10E-11
	GO:0031974	C	membrane-enclosed lumen	2.80E-12	2.30E-11
	GO:0044464	C	cell part	1.80E-08	1.30E-07
	GO:0005623	C	cell	1.80E-08	1.30E-07
	GO:0016020	C	membrane	7.70E-07	5.60E-06
Down-Regulated	GO:0042545	P	cell wall modification	2.20E-06	0.00088
	GO:0009664	P	plant-type cell wall organization	1.40E-06	0.00088
	GO:0044262	P	cellular carbohydrate metabolic process	4.70E-06	0.001
	GO:0005975	P	carbohydrate metabolic process	5.00E-06	0.001
	GO:0005976	P	polysaccharide metabolic process	9.60E-06	0.0016
	GO:0006260	P	DNA replication	1.60E-05	0.0021
	GO:0009827	P	plant-type cell wall modification	2.70E-05	0.0032
	GO:0060918	P	auxin transport	5.70E-05	0.0055
	GO:0009914	P	hormone transport	6.20E-05	0.0055
	GO:0003824	F	catalytic activity	1.30E-06	0.00031
	GO:0016757	F	transferase activity, transferring glycosyl groups	7.00E-05	0.0075
	GO:0016758	F	transferase activity, transferring hexosyl groups	9.50E-05	0.0075
	GO:0030312	C	external encapsulating structure	6.70E-07	4.70E-05
	GO:0005618	C	cell wall	5.90E-07	4.70E-05
	GO:0031225	C	anchored to membrane	9.00E-05	0.0042

In the table the Gene Ontology (GO) terms that are significantly (q ≤ 0.05) overrepresented using AgriGO V2 are observed. P corresponds to Biological Process, C corresponds to Cellular Component, and F corresponds to Molecular Function

### Functional association networks of DEGs at 2 dpi

**Fig. 5:**
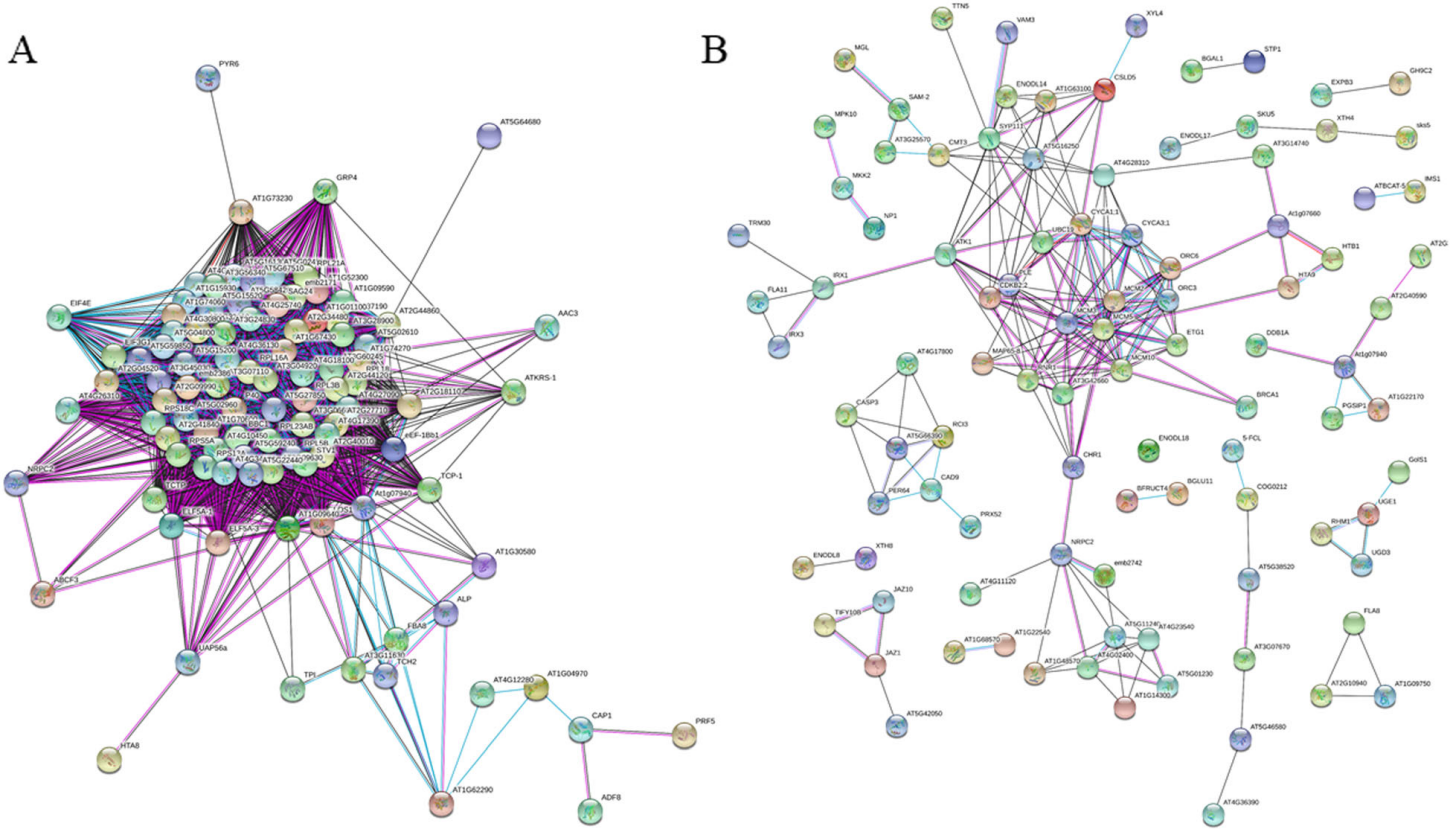
Functional association networks among DEGs corresponding to 2 dpi. **a** Interactions among the up-regulated genes obtained with the STRING software [35]. **b** Interactions among the down-regulated genes. Colored lines between nodes indicate the various types of interaction: black line, co-expression; light blue line, association in curated databases; purple line, experimental

Since the functional categorization suggested that DEGs coding for protein-related processes are the most contrasting categories when datasets of 2 and 10 dpi are compared, further inspection was carried out for DEGs at 2 dpi. Since genes encode products that interact each other, a network was generated to look for relationships among DEGs at 2 dpi (Fig. 5). Briefly, out of 309 up-regulated genes at 2 dpi with an ortholog in the Arabidopsis genome, only 282 were recognized by String [38]. Accordingly, most of interactions observed in the network corresponded to experimental data (purple lines) (Fig. 5a). Particularly, a central network was formed, involving 98 genes (nodes), from which most of them (80 nodes) were related to translation (structural constituents of ribosome) (Fig. 5a). For example, genes encoding proteins such as Ribosomal protein S12/S23 (Rps12/s23), Ribosomal protein l24B (Rpl24B), Ribosomal protein s6 (Rps6), Ribosomal protein s13 (Rps13), among others, formed the central network (Additional file 12: Table S7). In addition to genes involved in translation, genes associated to development were also found (Additional file 12: Table S7). Among this group, *YODA* (*YDA*), *STEROL METHYLTRANSFERASE 1* (*SMT1*), *EMBRYO DEFECTIVE 2386* (*EMB2386*), *MATERNAL EFFECT EMBRYO ARREST 22* (*MEE22*), and others, were found (Additional file 12: Table S7).

On the other hand, for down-regulated genes at 2 dpi, 256 were recognized by String out of 294 submitted genes (Fig. 5b). Also, a central network (49 nodes) was obtained with genes involved mainly in cell cycle, DNA replication and cell wall organization (Fig. 5b) (Additional file 12: Table S7). In this case, genes encoding proteins such as Cyclin A1;1 (CycA1;1), Cyclin-dependent kinase B2 (CdkB2), Minichromosome maintenance 3 (Mcm3), Origin recognition complex subunit 3 (Orc3), Cellulose synthase-like protein D5 (Csld5), Cellulose synthase A catalytic subunit 8 (CesA8), Cellulose synthase A catalytic subunit 7 (CesA7), among others, clearly formed a central network (Fig. 5b) (Additional file 12: Table S7). Notably, these networks were exclusively for DEGs at 2 dpi, since DEGs corresponding to 10 dpi did not show a clear interaction (Additional file 11: Figure S5). In resume, the generation and visualization of relationships among DEGs at 2 dpi show significantly more interactions than expected. In the case of up-regulated genes, they are mainly associated to ribosome biogenesis and translation as well as in development, whereas down-regulated genes are involved in cell cycle, DNA replication and cell wall organization.

### Differential gene expression of ribosome-related proteins at 2 dpi

**Fig. 6:**
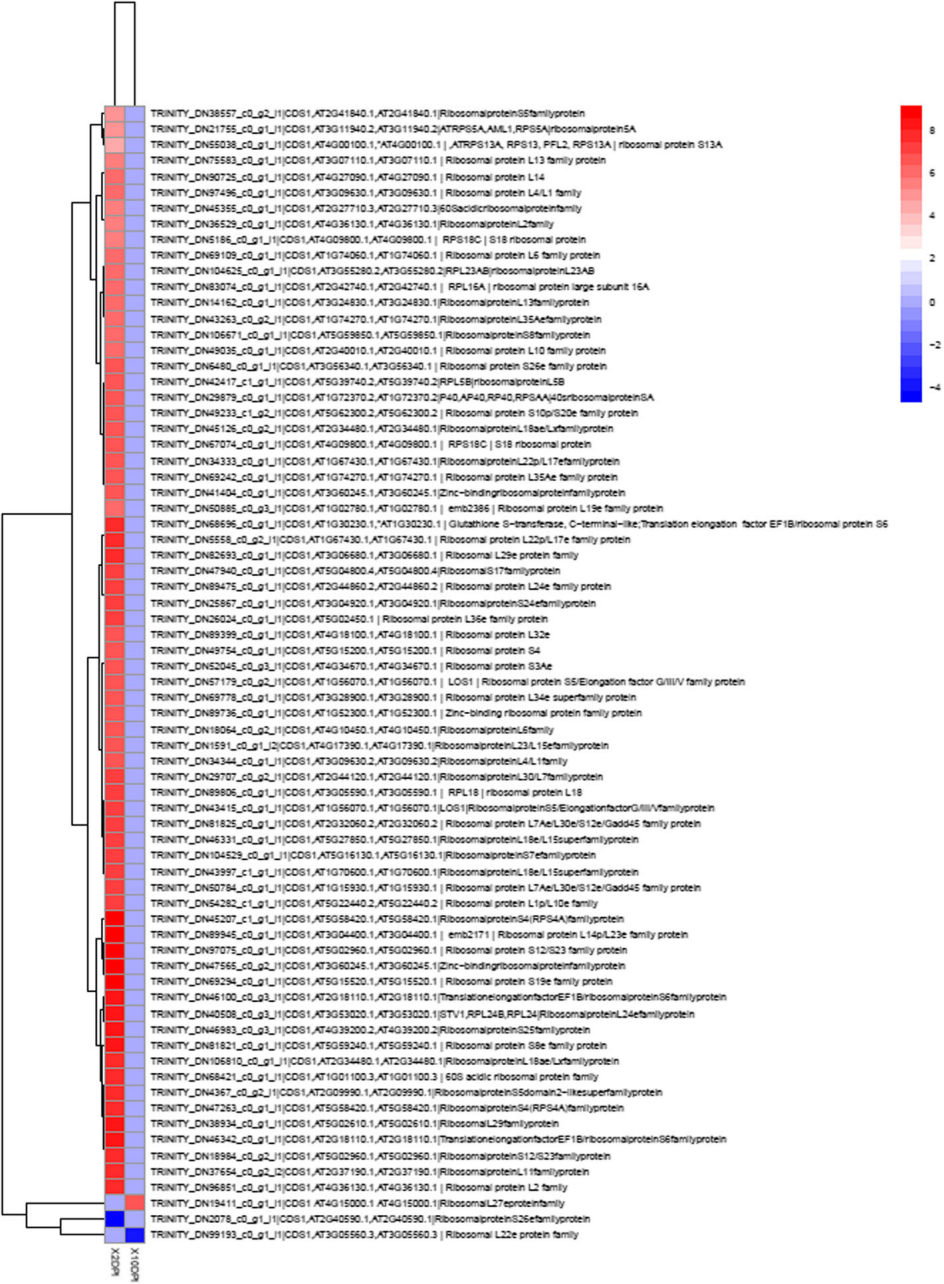
Heat map of ribosomal proteins comparing datasets of 2 and 10 dpi. The heat maps were made using the ggplot2 included in R package [36]

The finding that mainly proteins involved in ribosome biogenesis and translation (structural constituents of ribosome) were the most significant DEGs at 2 dpi, encouraged to focus on these genes. As observed in Fig. 6, proteins related to ribosome biogenesis and translation were significantly up-regulated in 2 dpi compared to 10 dpi, 72 of which were exclusively expressed in the treatment at 2 dpi. These exclusive genes corresponded to ribosomal proteins, for which a significant increase in their expression pattern was observed only at 2 dpi (Fig. 6). As mentioned before, ribosomal proteins such as Ribosomal protein s12/s23 (Rps12/S23), Ribosomal protein l24B (Rpl24B), Ribosomal protein s6 (Rps6), Ribosomal protein s13 (Rps13), among others, were found up-regulated at 2 dpi. In summary, ribosomal-related proteins are found up-regulated at 2 dpi, suggesting that translation is impacted upon infection by *Fov*.

## Discussion

As supported by several studies around the world, *Fov* is the principal species that causes RSR in vanilla plants [25, 39, 40]. Although the generation and use of resistant varieties are the best mean to restrict *Fov*, scarce information about the plant-pathogen interaction, as well as limited genetic resources, have impeded to eradicate or limit the devastation that cause *Fov* in vanilla production. Under such scenario, the understanding of mechanistic responses of vanilla plants upon infection by *Fov* is scarce and necessary. Therefore, the primary goal of this work was to elucidate the early and late mechanistic responses of vanilla plants induced by *Fov* through investigating whole transcriptional changes in root tissues (Fig. 1). The RNA-Seq technique was employed to detect the DEGs during two frame times of infection by this root-infecting fungal pathogen, namely at early (2 dpi) and late (10 dpi) stages. The RNA-Seq analysis carried out in roots revealed that 4480 and 881 genes were differentially modulated by *Fov* at 2 and 10 dpi, respectively, as compared to the control (Fig. 2). This result indicated that the major transcriptional change occurs at early stages of infection, encouraging further analysis for these DEGs (Fig. 3). After functional classification of DEGs at 2 and 10 dpi, it was further confirmed that only DEGs at 2 dpi contained enriched functional categories (Fig. 4). For instance, enrichment analyses revealed the involvement of DEGs at 2 dpi to ribosome biogenesis and translation for up-regulated genes, whereas down-regulated genes were mainly associated to cell wall biogenesis (Table 1).

Most biological processes, from cell differentiation to organ development, as well as the adaptation to the environment, relies on transcriptional adjustments. Even that gene expression regulation is solidly established, it is clear that regulation beyond this level also plays a pivotal role in modulating key biological processes. Among the enriched functional categories for DEGs at 2 dpi, translation was the most prominent among up-regulated genes (Table 1) (Additional file 10: Figure S4), suggesting that this biological process is significantly impacted upon infection by *Fov*. Moreover, the formation a single network involving all these RPs supports a putative function in the early stages of infection by *Fov* (Fig. 5a). On the other hand, the finding that down-regulated genes are mostly involved in cell wall modifications (Table 1), is in agreement with the known susceptibility of *V. planifolia* plants to *Fov*. In this regard, since the plant cell wall acts as an important barrier against pathogen penetration by activating cell wall strengthening-related genes [41], the down-regulation of these genes reflects the facilitation of pathogen entry and then the negative impact on processes such as cell division and DNA replication of plant cells (Fig. 5b).

Being the basic infrastructure for protein translation, ribosomal proteins (RPs) have been known primarily for their housekeeping functions [42]. However, in the recent years, emerging functions of RPs have been described, including regulation of gene expression through translational mechanisms [43, 44]. One hint for this is, for example, that even there are at least 230 genes encoding RPs in the Arabidopsis genome, a single member of each family of RPs has been found as part of the subunits of ribosomes, suggesting that expression of the additional RPs are subjected to different cues, including environmental conditions [45, 46]. Among the up-regulated RPs found in this work, *RPL13* has been related to the tolerance of potato to *Verticullum dahliae* [47]. Similarly, *RPL10*, *RPS12/S23*, and *PRPL19e* [48, 49], as well as the expression of *RPS6*, *RPL19*, *RPL7*, and *RPS2* [50], have been associated to plant response against bacteria and virus; respectively. Also, *RPS10* and *RPS10p/S20e* have been found to be up-regulated by *Phytophthora sojae* in *Glycine max* [51]. Finally, *RPL12* and *RPL19* also have been shown to participate in the resistance against *P. syringae* in *Nicotiana benthamiana* and *A. thaliana*, respectively [49, 52]. On the other hand, abiotic stress has also been found to induce transcription of RPs. For example, transcript levels of *RPS15a* (and its variants A, C, D and F) increased significantly in response to heat stress in Arabidopsis [53]. This was also the case for *RPS14*, *RPL13*, and *RPL30* in Arabidopsis, in which their expression levels augment under the treatment with benzylaminopurine [54]. In addition, *RPL10* and *RPL10C* were induced when Arabidopsis plants were treated with UV, like those results obtained in maize plants [55, 56]. Finally, regarding low temperature conditions, increase of *RPS6*, *RPS13* and *RPL37* have been observed in *Glycine max* and *Brassica napus* [57, 58]. Besides the association of these RPs to biotic or abiotic stresses, functional characterization of them has allowed to elucidate their role in plants. In that sense, mutation of *RPL10* causes lethality of the female gametophyte in Arabidopsis [59]. Also, the mutation of *RPS13A* results in a reduction of cell division, retardation of flowering, and delayed growth of shoots and leaves [60]. Similar phenotypes of growth retardation and fertility reduction have been reported in the *RPL23aA* mutant [61]. In summary, until now, the central role of RPs in development as their global participation in response to abiotic stress in iron and phosphate deficit conditions has been assessed. Here, we report for the first time the RPs global participation in response to biotic stress in a translational manner.

**Fig. 7:**
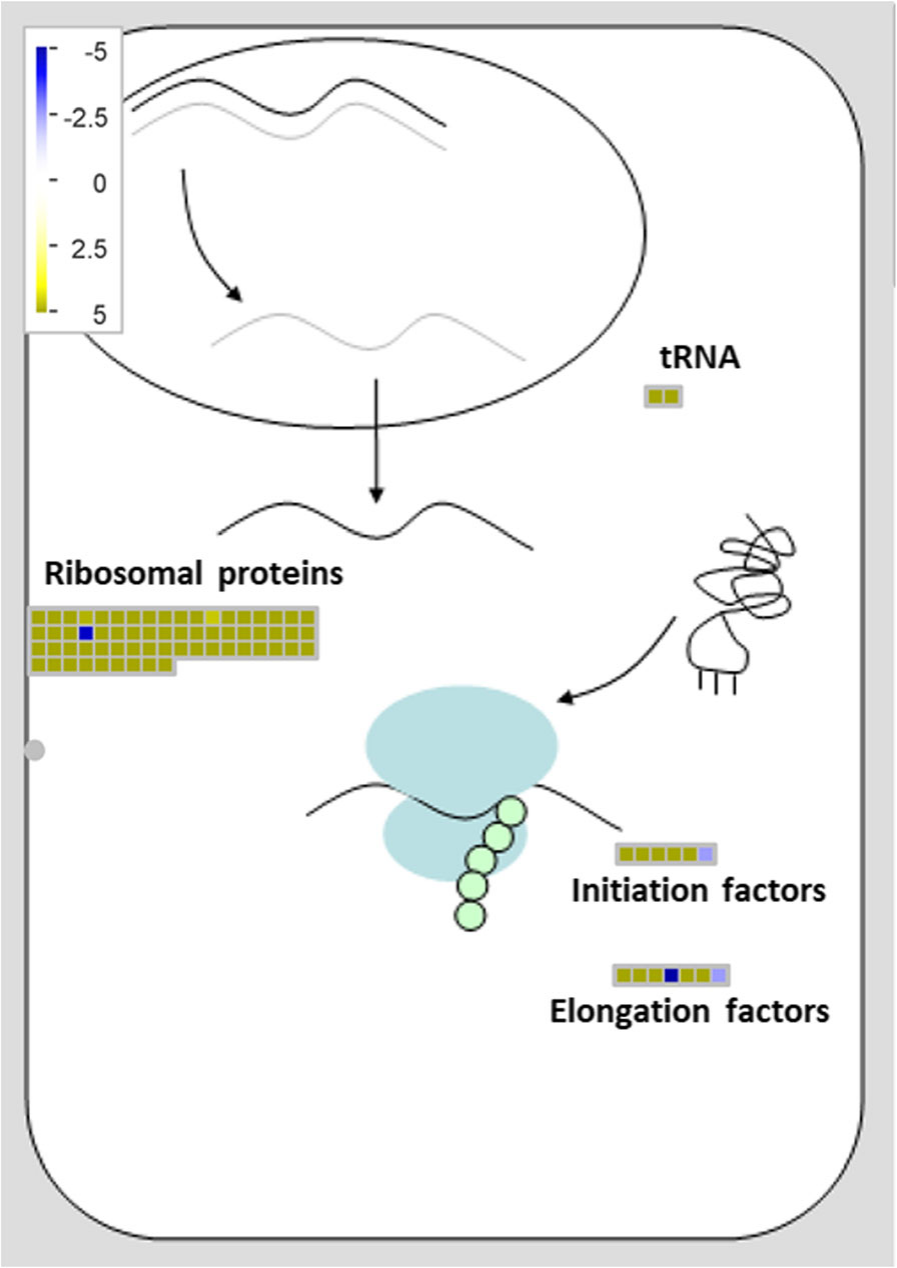
Overview of MapMan RNA-protein synthesis at 2 dpi. Transcript levels of translation-related genes (RPs, tRNAs, initiation factors and elongation factors) are shown. Upon invasion by *Fov*, induction of RPs leads to changes in ribosome composition, driving to selective translation of certain transcripts

Since translation of proteins is energetically a demanding process, stress can cause a global drop of protein synthesis in plants. However, a translational regulation mechanism leading to the translation of certain transcripts to produce specific RPs may be the key to the survival of plants under stressful conditions [42]. Only in recent years this kind of regulation beyond the transcriptional level has received special interest due to its implications in key biological processes, particularly those related to biotic and abiotic stress responses [43, 44, 62]. Thus, the up-regulation of several RPs during early stages of infection by *Fov* in vanilla plants represent a whole response of translational regulation (Fig. 7), as known about of regulation of translational factors and their associated proteins with translational regulation [62]. In the transcriptional profile of vanilla plants at 2 dpi, induction of *RPL24B*, *RPS18*, *RPS5* and *RPL27A* were found. Since *RPL24B* of Arabidopsis is related to the translation regulation of some auxin signaling genes that contains uORFs [63, 64], the up-regulation of this RP in vanilla plants could suggest the presence of a similar mechanism. Supporting this hypothesis, mutants of *RPL24B*, as well as *RPS18A*, *RPS5B*, *RPS13B* and *RPL27A*, known as “pointed first leaf” mutants, show defective phenotypes related to development, such as the reduction of the growth of shoots and roots [60, 65–67]. This suggests that, these proteins carry out specific functions during plant development, likely by translating specific transcripts. Accordingly, RPS6, which is responsive to biotic and abiotic stresses, is also a regulator of translation [68]. Specifically, phosphorylation of RPS6 through the TOR signaling pathway lead to the selective translation of mRNAs [42, 62]. Besides RPS6 phosphorylation, phosphorylation of eIF2α by the Gcn2-Gcn1 complex reduces global protein synthesis, which has implications for growth and development [69–73]. Since *GCN1*, *GCN2* and *EIF2α* were exclusively found in DEGs at 2 dpi and its expression resulted to be high (near to 12 log2 fold change), this suggest, same as in abiotic and biotic stress [74], that the general translation can be decreased, accompanied by selective translation through changes of ribosome composition as the response to pathogen infection.

Under such scenario, upon infection by *Fov*, root cells of vanilla plants likely change the expression of RPs, resulting in alterations of ribosomes composition, as reported against abiotic stress [75] and, therefore, in modulation of translation for certain transcripts as a response of fungal invasion (Fig. 7). This is particularly relevant since this is the first time that RPs are associated to *Fov*-derived response in vanilla plants. Moreover, it has been indicated that the typical chromosomal number of *V. planifolia* is 2n = 32 and more recently, cytogenetic studies conducted in the Mansa morphotype, reported an intra-individual variation in the number of chromosomes in the apical cells of the root, which may vary from 2n = 20 to 2n = 32 or more. Likewise, the existence of a “progressively partial endoreplication” in *V. planifolia* has been reported, however, this process does not occur in all tissues and some studies have reported that less than half of the genome of *V. planifolia* is being replicating effectively in each cycle. However, we consider that the methodology used in this study minimizes the effect of this phenomenon, so we propose the role of the translational regulation in the early plant response in the interaction with pathogen [76]*.*

## Conclusions

The screening of transcriptional changes of *V. planifolia* upon infection by *F. oxysporum* f. sp. *vanillae* shows that the major change occurs at early stages of infection, according to the analysis of DEGs at 2 dpi that shows, among other biological processes, the transcription of RPs increases specifically at this moment. Moreover, given the changes of these RPs are involved in plant developmental programs, as well as in response to biotic and abiotic stress conditions, their differential expression point to a biological role during infection. Therefore, is proposed that in response to *Fov* infection, root cells of vanilla plants activate a transcriptional reprogramming coupled with a translational regulation. The results presented here highlight key processes and potential molecular players that might be further studied to develop vanilla breeding programs, help to fight the most damaging disease of this crop.

## Methods

### Plant material

From plants of *V. planifolia* Jacks. (Mansa morphotype) growing on a farm located in the Totonacapan region (Veracruz, Mexico), samples were collected and propagated under greenhouse conditions. Vigorous and pathogen-free plants were used in the present study at the developmental age of 12 weeks for infectivity assays. Such plants exhibited leaf morphology characteristic of *V. planifolia*. Sixty plants were distributed in twelve groups of five each one, for an experimental design intended for four treatments (two times conditions and two control) and three biological replicates by treatment (Fig. 1). The time conditions were 2 dpi (2 days post-inoculate) and 10 dpi (10 days post-inoculate) and the controls were plants non-treated with *Fov*.

### Infectivity assays

The in vitro fungal infection of *V. planifolia* plants was carried out with the JAGH3 strain of *Fov*. This strain of *Fov* was isolated from *V. planifolia* (Mansa morphotype) with evident RSR [77], its pathogenic capacity was proven, so it has been used for further studies [25, 40, 78]. Briefly, cuttings of *V. planifolia* were subjected to darkness for ten days. The absence of light exposition allowed the generation of new roots. A mechanical incision was made in each root under aseptic conditions. Then, roots were exposed to an aqueous solution of spores with a concentration of 1 × 10^6^ CFU of *Fov* (JAGH3 strain). The inoculation was carried out directly on the substrate where cuttings were established. Cuttings belonging to the control group were treated similarly, exposing them to an aqueous solution free of spores. For a single biological experiment, control and treatment experiments consisted of 30 plants of the same age, established on substrate and maintained under greenhouse conditions with a 12-h photoperiod (shaded). Two biological experiments were carried out covering two frames of time of *Fov* infection, namely 2 and 10 days post-inoculation (dpi) (Fig. 1). For each of the treatments and their respective controls, five tissue samples were collected in each case, pooled and processed immediately for RNA extraction. In total, twelve pools were obtained, covering two biological experiments for each treatment time (2 and 10 dpi).

The pathogenicity of the JAGH3 strain on vanilla plants was evaluated following the protocol specified by Koyyappurath et al., (2015) [79]; which is based on the observation and recording of infection symptoms, on the alternate days after inoculation. According to the above, the symptoms were observed and recorded on alternate days in aerial parts, which include aerial roots, in addition to the stem and leaves; The observation period was from day 1 to day 9, after inoculation. The presence of the characteristic symptoms of the infection was monitored using a rating scale of 0–4 as follows: 0 = no symptoms; 1 = the leaves lost their brightness; 2 = local browning visible on the stem; 3 = lodging of plants, brown areas and mycelium visible in the aerial parts; and 4 = totally rotten or dead plant.

### Total RNA extraction

For the total RNA extraction from the roots of vanilla plants, a protocol was standardized based on a previous report [80]. Briefly, 200 mg of root tissue were homogenized with the Trizol reagent and then treated with Phenol:Chloroform:Isoamyl Alcohol (25:24:1), followed by vortexing and centrifugation. The upper aqueous phase was transferred into silica columns included in the SV Total RNA Isolation System extraction kit from Promega. The integrity of the obtained RNA was determined by electrophoresis in 2% agarose gel, stained with ethidium bromide (EtBr 0.5 μg ml^− 1^) under denaturing conditions. The concentration of total RNA samples was verified using a NanoDrop spectrophotometer, as well as its RNA Integrity Number (RIN) values were obtained with an Agilent 2100 Bioanalyzer system (Agilent Technologies). RNA samples with RIN values > 6 were used for cDNA synthesis and subsequent sequencing.

### Generation and sequencing of cDNA libraries

The generation and sequencing of the cDNA libraries was carried out in the University Unit of Massive Sequencing and Bioinformatics of the Institute of Biotechnology of the National Autonomous University of Mexico (UUSMB IBT-UNAM). In total, the construction of 12 cDNA libraries was carried out. Afterwards, the sequencing of the cDNA libraries was performed using the Nextseq 500 illumina platform, generating paired-end reads of 76 bp. In total, 204 million 517 thousand 080 reads were obtained.

### De novo transcriptome assembly and annotation

Quality of reads obtained from the high-throughput sequencing was carried out using the FastQC software (https://www.bioinformatics.babraham.ac.uk/projects/fastqc/). Reads above 32 nt, without the presence of adapters were considered for further analysis. First, to filter and discard reads corresponding to the plant pathogen used in the infectivity assays, alignment of reads was performed with the Smalt software (version 0.7.6) using the reference genome of *F. oxysporum* f. sp. *lycopersici* strain Fol4287. Then, de novo transcriptome assembly corresponding to *V. planifolia* reads was made using the Trinity software (version 2.4). For assessing the quality of the obtained transcriptome assembly, metrics like total number of contigs, longest contig length, mean and median contig length, and N50 were calculated using TransRate, followed by an analysis with BUSCO to explore completeness according to conserved ortholog content. The analysis with the BUSCO software was carried out using the Liliopsida odb10* database, following the software default parameters [81]. Subsequently, the annotation of the transcriptions was made with the Trinotate software. The search for the open reading frames in the transcriptions was made with the TransDecoder software. Transcripts and amino acid sequences were aligned against the UniProt database using Blastn and Blastx. Moreover, the presence of PFAM domains in the protein sequences predicted from the transcripts was tested with the HMMER software. Finally, the annotation of the transcripts was done using Blast2go [36], as well as the databases of Gene Ontology (GO), KEGG, COG.

### Differential expression analysis and functional categorization

For assessing differentially expressed genes (DEGs) of the assembled transcripts, a method based on mapping the reads against the assembled transcriptome was done. Such mapping of reads was done with Bowtie2, as part of the Trinity pipeline, followed by an analysis with RSEM. The results obtained by RSEM were submitted to IDEAMEX [82], a website intended for differential expression analysis using several approaches. Specifically, IDEAMEX analysis is based on DESeq [83], DESeq2 [84], NOISeq [85] and EdgeR [86] methods. For selection of DEGs, the following parameters were used: padj <= 0.04, FDR < = 0.04 and prob.> = 0.96, and a logFC> = 2. Heatmaps for DEGs were done in R using the ggplot2 package [87]. From the functional annotation of the assembled transcripts obtained by Blast2GO, visualization of the transcriptome regarding expression patterns was performed with Mapman V2 software [37]. For functional categorization, DEGs were submitted to the agriGO v2.0 software [88], selecting the singular enrichment analysis (SEA). The enrichment analysis was carried out according to the following parameters in the AgriGo v2.0 Software, Statistical test method Fisher; Multi test adjustment method: Yekutieli (FDR under dependency); and 0.05 of significance level. The enrichment analysis was also carried out with PANTHER classification system software (v.14.0). under the following specifications, statistical test method Fisher; Multi test adjustment method: Bonferroni; and 0.05 of significance level. Finally, gene networks among DEGs were obtained with the STRING software [38].

## Supplementary information

**Additional file 1: Figure S1.** PCA graph of 2 dpi treatment and control treatment. The graph analyzes the spatial dispersion between treatment and control and their respective replicas.

**Additional file 2: Figure S2.** PCA graph of 10 dpi treatment and control treatment. The graph analyzes the spatial dispersion between treatment and control and their respective replicas.

**Additional file 3: Table S1.** Statistics of sequencing and filtering of raw data.

**Additional file 4: Figure S3.** Annotation all unigenes derived from the de novo transcriptome assembly of *V. planifolia* upon infection by *Fov*. Annotation was based on Gene Ontology terms using Blast2GO. GO categories are as follow: biological process (BP), molecular function (MF), and cellular component (CC). The number of genes corresponding to each functional category is shown.

**Additional file 5: Table S2.** Assembly statistics and sequence mapping. (XLSX 12 kb)

**Additional file 6: Table S3.** DEGs at 2 and 10 dpi obtained with several methods, including DESeq, DESeq2, NOISeq and EdgeR.

**Additional file 7: Table S4.** Functional annotation of DEGs at 2 and 10 dpi obtained with the EdgeR method.

**Additional file 8: Table S5.** GO terms enrichment analysis, performed with the PANTHER classification system software (v.14.0). Gene Ontology categories significantly enriched in DEGs at 2 dpi, present in transcripts up regulated, in the infection caused by Fusarium in vanilla. In the table the Gene Ontology (GO) terms that are significantly (q ≤ 0.05) overrepresented using are observed.

**Additional file 9: Table S6.** GO terms enrichment analysis, performed with the PANTHER classification system software (v.14.0). Gene Ontology categories significantly enriched in DEGs at 2 dpi, present in transcripts down regulated, in the infection caused by Fusarium in vanilla. In the table the Gene Ontology (GO) terms that are significantly (q ≤ 0.05) overrepresented using are observed.

**Additional file 10: Figure S4.** Schematic representation of biological processes enriched in DEGs at 2 dpi. a Biological processes enriched among up-regulated genes. b Biological processes enriched among down-regulated genes. Enrichment analysis was performed with agriGO. Enriched GO terms considered as significant are indicated by corresponding color levels.

**Additional file 11: Figure S5.** Functional association networks among DEGs at 10 dpi. a Interactions among the up-regulated genes. b Interactions among the down-regulated genes. Colored lines between nodes indicate the various types of interaction: black line, co-expression; light blue line, association in curated databases; purple line, experimental.

**Additional file 12: Table S7.** In this table, the genes corresponding to the nodes of the network of genetic interactions, obtained with the string software, are observed. This analysis corresponds to the genes differentially expressed at 2dpi, in the infection caused by *Fusarium* in vanilla. Genes (nodes) composing the central networks shown in Fig. 5.

## Data Availability

The datasets used and/or analyzed during the current study are available from the corresponding author on reasonable request and they were submitted to the GEO platform of NCBI-GenBank (Accession number: GSE134155).

## Abbreviations

eiFs: Eukaryotic initiation factors
GCN: General control non-derepressible
logFC: log2 of fold change
RNA-Seq: High-throughput sequencing of RNA
RPL: Large subunit of ribosomal protein
RPS: Small subunit of ribosomal protein
