# Supplementary figures and images for: Functional categorization of de novo transcriptome assembly of Vanilla planifolia Jacks. potentially points to a translational regulation during early stages of infection by Fusarium oxysporum f. sp. vanillae
Source: BMC Genomics. 2019 Nov 8;20:826. doi: 10.1186/s12864-019-6229-5 (PMC6839141; doi:10.1186/s12864-019-6229-5)

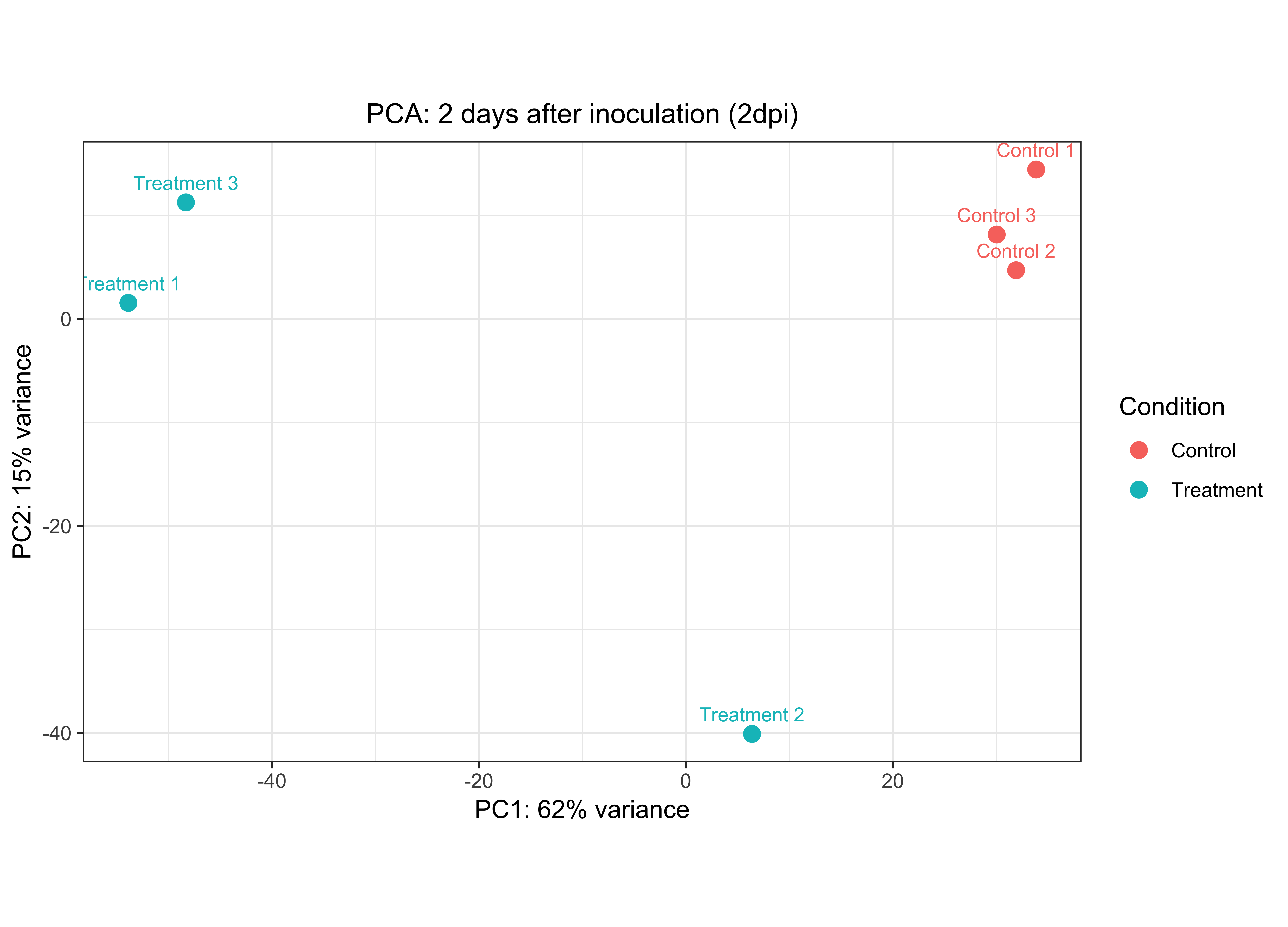

Supplement: Supplementary file 1 — Additional file 1: Figure S1. PCA graph of 2 dpi treatment and control treatment. The graph analyzes the spatial dispersion between treatment and control and their respective replicas. [file 12864_2019_6229_MOESM1_ESM.png]

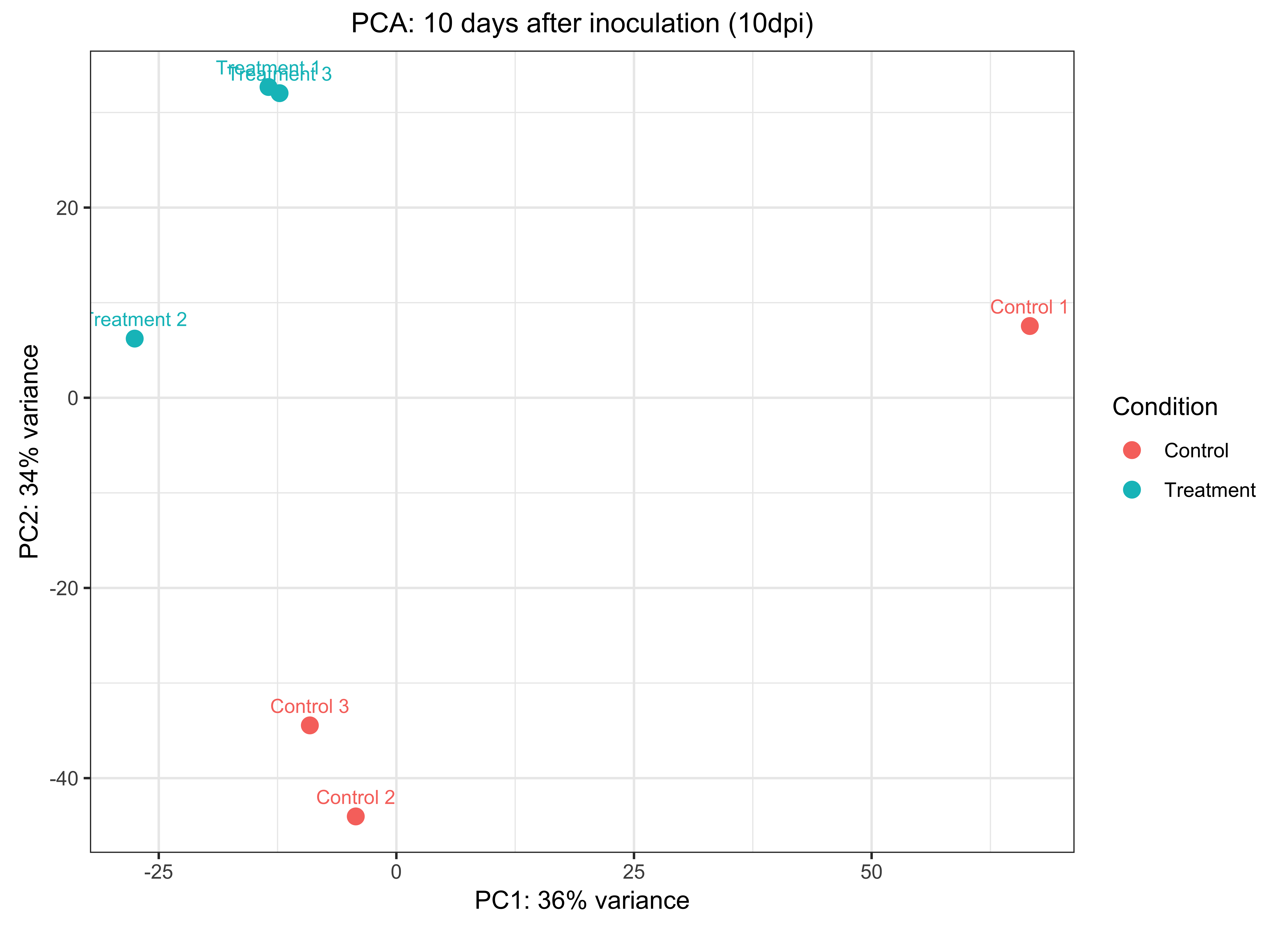

Supplement: Supplementary file 2 — Additional file 2: Figure S2. PCA graph of 10 dpi treatment and control treatment. The graph analyzes the spatial dispersion between treatment and control and their respective replicas. [file 12864_2019_6229_MOESM2_ESM.png]

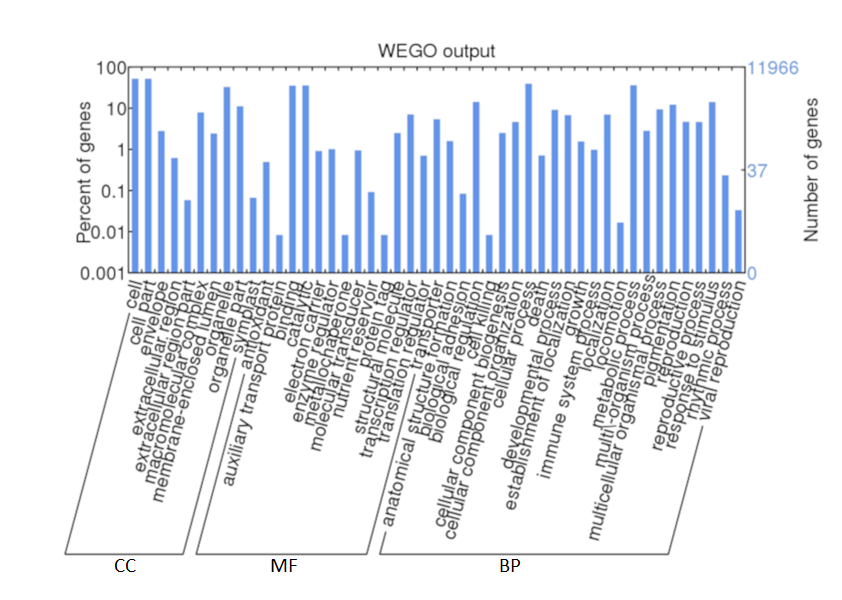

Supplement: Supplementary file 4 — Additional file 4: Figure S3. Annotation all unigenes derived from the de novo transcriptome assembly of V. planifolia upon infection by Fov. Annotation was based on Gene Ontology terms using Blast2GO. GO categories are as follow: biological process (BP), molecular function (MF), and cellular component (CC). The number of genes corresponding to each functional category is shown. [file 12864_2019_6229_MOESM4_ESM.png]

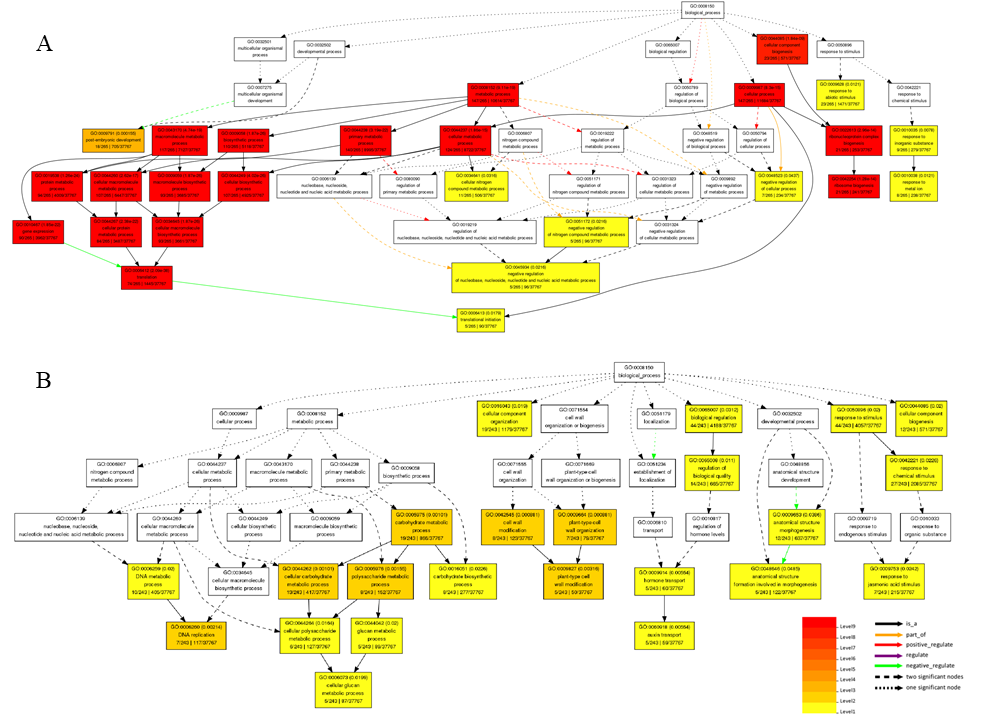

Supplement: Supplementary file 10 — Additional file 10: Figure S4. Schematic representation of biological processes enriched in DEGs at 2 dpi. a Biological processes enriched among up-regulated genes. b Biological processes enriched among down-regulated genes. Enrichment analysis was performed with agriGO. Enriched GO terms considered as significant are indicated by corresponding color levels. [file 12864_2019_6229_MOESM10_ESM.png]

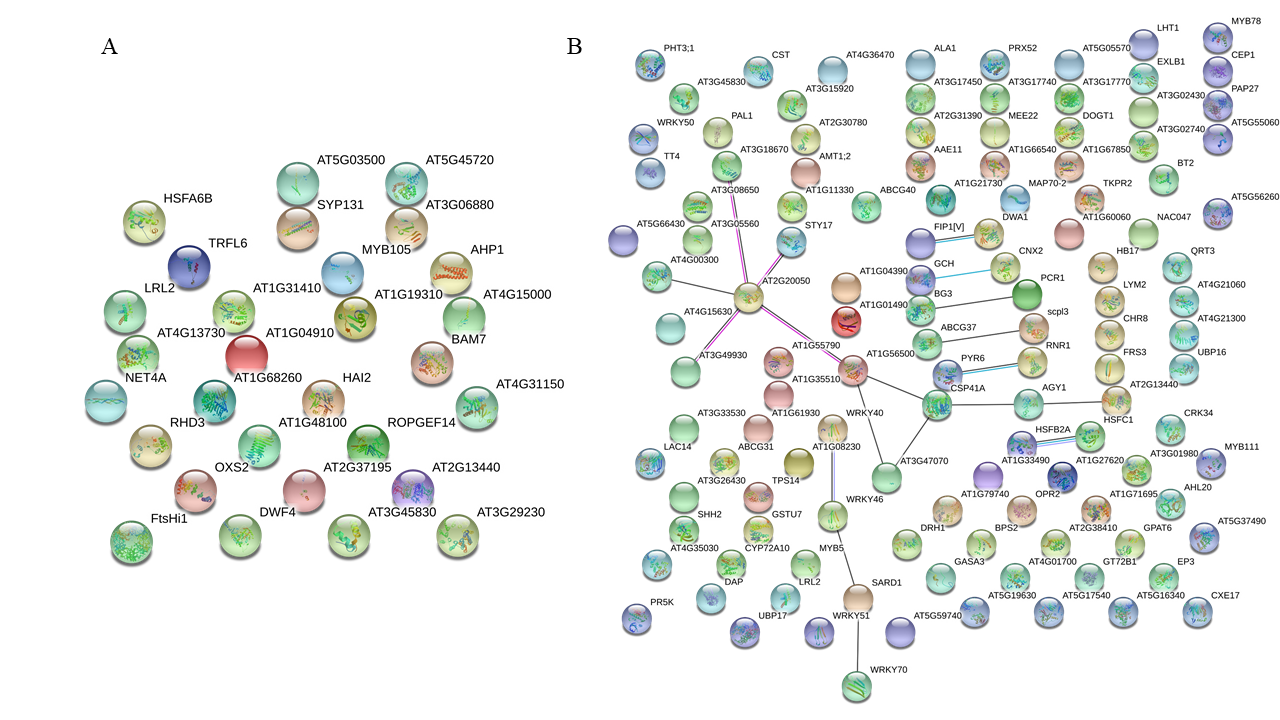

Supplement: Supplementary file 11 — Additional file 11: Figure S5. Functional association networks among DEGs at 10 dpi. a Interactions among the up-regulated genes. b Interactions among the down-regulated genes. Colored lines between nodes indicate the various types of interaction: black line, co-expression; light blue line, association in curated databases; purple line, experimental. [file 12864_2019_6229_MOESM11_ESM.png]
