# Supplementary material for: Functional categorization of de novo transcriptome assembly of Vanilla planifolia Jacks. potentially points to a translational regulation during early stages of infection by Fusarium oxysporum f. sp. vanillae
Source: BMC Genomics. 2019 Nov 8;20:826. doi: 10.1186/s12864-019-6229-5 (PMC6839141; doi:10.1186/s12864-019-6229-5)
Supplement: Supplementary file 12 — Additional file 12: Table S7. In this table, the genes corresponding to the nodes of the network of genetic interactions, obtained with the string software, are observed. This analysis corresponds to the genes differentially expressed at 2dpi, in the infection caused by Fusarium in vanilla. Genes (nodes) composing the central networks shown in Fig. 5. [file 12864_2019_6229_MOESM12_ESM.docx]

| DEGs | Protein Name | Protein Identifier | Protein Description |
| --- | --- | --- | --- |
| Up-Regulated | emb2386 | AT1G02780.1 | Ribosomal protein L19e family protein; Embryo defective 2386 (emb2386) |
|  | AT4G39200 | AT4G39200.1 | Ribosomal protein S25 family protein |
|  | BBC1 | AT3G49010.3 | Encodes 60S ribosomal protein L13 |
|  | AT1G01100 | AT1G01100.2 | 60S acidic ribosomal protein family |
|  | AT2G09990 | AT2G09990.1 | Ribosomal protein S5 domain 2-like superfamily protein |
|  | AT5G15520 | AT5G15520.1 | Ribosomal protein S19e family protein |
|  | AT5G02610 | AT5G02610.2 | Ribosomal L29 family protein |
|  | AT4G10450 | AT4G10450.1 | Ribosomal protein L6 family |
|  | SAG24 | AT1G66580.1 | Senescence associated gene 24 (SAG24) |
|  | PHT3;1 | AT5G14040.1 | Mitochondrial phosphate carrier protein 3, mitochondrial |
|  | AT3G56340 | AT3G56340.1 | Ribosomal protein S26e family protein |
|  | AT4G27090 | AT4G27090.1 | Ribosomal protein L14 |
|  | ELF5A-1 | AT1G13950.1 | Encodes eukaryotic translation initiation factor 5A (EIF-5A) |
|  | AT5G02960 | AT5G02960.1 | Ribosomal protein S12/S23 family protein |
|  | AT4G30800 | AT4G30800.1 | Nucleic acid-binding, OB-fold-like protein |
|  | STV1 | AT3G53020.1 | RPL24B encodes ribosomal protein L24 |
|  | AT3G07110 | AT3G07110.2 | Ribosomal protein L13 family protein |
|  | AT5G16130 | AT5G16130.1 | Ribosomal protein S7e family protein |
|  | RPL5B | AT5G39740.2 | 60S ribosomal protein L5-2; Component of the ribosome |
|  | AT3G45030 | AT5G62300.1 | Ribosomal protein S10p/S20e family protein |
|  | AT5G02450 | AT5G02450.1 | Ribosomal protein L36e family protein |
|  | AT1G52300 | AT1G52300.1 | Zinc-binding ribosomal protein family protein |
|  | AT1G15930 | AT1G15930.1 | Ribosomal protein L7Ae/L30e/S12e/Gadd45 family protein |
|  | AT2G41840 | AT2G41840.1 | Ribosomal protein S5 family protein |
|  | AT3G28900 | AT3G28900.1 | Ribosomal protein L34e superfamily protein |
|  | AT2G32060 | AT2G32060.1 | Ribosomal protein L7Ae/L30e/S12e/Gadd45 family protein |
|  | AT2G40010 | AT2G40010.1 | Ribosomal protein L10 family protein |
|  | AT2G44120 | AT2G44120.2 | Ribosomal protein L30/L7 family protein |
|  | AT1G74270 | AT1G74270.1 | Ribosomal protein L35Ae family protein |
|  | AT3G06680 | AT3G06680.1 | Ribosomal L29e protein family |
|  | AT5G59850 | AT5G59850.1 | Ribosomal protein S8 family protein |
|  | RPS18C | AT4G09800.1 | 40S ribosomal protein S18 |
|  | RPS5A | AT3G11940.1 | One of two genes encoding the ribosomal protein S5 |
|  | AT4G17390 | AT4G17390.1 | Ribosomal protein L23/L15e family |
|  | AT4G34670 | AT4G34670.1 | Ribosomal protein S3Ae |
|  | AT4G36130 | AT4G36130.1 | Ribosomal protein L2 family |
|  | P40 | AT1G72370.1 | 40S ribosomal protein Sa-1 |
|  | GRP4 | AT3G23830.2 | Glycine-rich RNA-binding protein 4, mitochondrial |
|  | RPL3B | AT1G61580.1 | 60S ribosomal protein L3-2; R-protein L3 B (RPL3B) |
|  | EIF3G1 | AT3G11400.2 | Eukaryotic translation initiation factor 3 subunit G |
|  | AT3G60245 | AT3G60245.1 | Zinc-binding ribosomal protein family protein |
|  | RPS13A | AT4G00100.1 | Encodes a cytoplasmic ribosomal protein S13 homologue |
|  | AT2G04520 | AT2G04520.1 | Putative translation initiation factor eIF-1A |
|  | AT2G37190 | AT2G37190.1 | Ribosomal protein L11 family protein; Binds directly to 26S ribosomal RNA |
|  | EIF4E | AT4G18040.1 | Eukaryotic translation initiation factor 4E-1 |
|  | AT5G15200 | AT5G15200.1 | Ribosomal protein S4 |
|  | AT5G58420 | AT5G58420.1 | Ribosomal protein S4 (RPS4A) family protein |
|  | emb2171 | AT3G04400.1 | Ribosomal protein L14p/L23e family protein; Embryo defective 2171 (emb2171) |
|  | AT3G09630 | AT3G09630.1 | Ribosomal protein L4/L1 family |
|  | AT5G22440 | AT5G22440.2 | Ribosomal protein L1p/L10e family |
|  | NRPC2 | AT5G45140.1 | DNA-directed RNA polymerase III subunit 2 |
|  | RPL21A | AT1G09590.1 | Translation protein SH3-like family protein |
|  | LOS1 | AT1G56070.1 | Ribosomal protein S5/Elongation factor G/III/V family protein |
|  | TRX1 | AT3G51030.1 | Thioredoxin H-type 1 |
|  | AT3G04920 | AT3G04920.1 | Ribosomal protein S24e family protein |
|  | AT2G44860 | AT2G44860.1 | Probable ribosome biogenesis protein RLP24 |
|  | AT3G24830 | AT3G24830.1 | Ribosomal protein L13 family protein |
|  | AT1G67430 | AT1G67430.1 | Ribosomal protein L22p/L17e family protein |
|  | AT5G59240 | AT5G59240.1 | Ribosomal protein S8e family protein |
|  | AT2G27710 | AT2G27710.1 | 60S acidic ribosomal protein family |
|  | AT4G18100 | AT4G18100.1 | Ribosomal protein L32e; Involved in translation, ribosome biogenesis |
|  | AT5G04800 | AT5G04800.4 | Ribosomal S17 family protein |
|  | RPL18 | AT3G05590.1 | Encodes cytoplasmic ribosomal protein L18 |
|  | RPL16A | AT2G42740.1 | Ribosomal protein large subunit 16A |
|  | AT5G67510 | AT5G67510.1 | Translation protein SH3-like family protein |
|  | AT1G70600 | AT1G70600.1 | Ribosomal protein L18e/L15 superfamily |
|  | AT1G74060 | AT1G74060.1 | Ribosomal protein L6 family protein |
|  | RPL23AB | AT3G55280.1 | 60S ribosomal protein L23A (RPL23aB). Paralog of RPL23aA |
|  | ELF5A-3 | AT1G69410.1 | Eukaryotic translation initiation factor 5A-3 |
|  | AT2G34480 | AT2G34480.1 | Ribosomal protein L18ae/LX family protein |
|  | AT5G27850 | AT5G27850.1 | Ribosomal protein L18e/L15 superfamily protein |
|  | AT1G73230 | AT1G73230.1 | Nascent polypeptide-associated complex NAC |
|  | AT4G25740 | AT4G25740.1 | RNA binding Plectin/S10 domain-containing protein |
|  | AT1G09640 | AT1G09640.1 | Translation elongation factor EF1B, gamma chain |
|  | TCTP | AT3G16640.1 | Encodes a protein homologous to translationally controlled tumor protein (TCTP) from Drosophila |
|  | ABCF3 | AT1G64550.1 | Member of GCN subfamily; Belongs to the ABC transporter superfamily. |
|  | AT1G07920 | AT1G07930.1 | GTP binding Elongation factor Tu family protein |
|  | AAC3 | AT4G28390.1 | Encodes a mitochondrial ADP/ATP carrier protein |
|  | WEE1 | AT1G02970.1 | Wee1-like protein kinase |
|  | AT1G30580 | AT1G30580.1 | Obg-like ATPase 1; Hydrolyzes ATP, and can also hydrolyze GTP with lower efficiency |
|  | UAP56a | AT5G11170.1 | DEAD/DEAH box RNA helicase family protein |
|  | AT4G26310 | AT4G26310.2 | Elongation factor P (EF-P) family protein |
|  | TPI | AT3G55440.1 | Triosephosphate isomerase, cytosolic; Encodes triosephosphate isomerase |
|  | TCP-1 | AT3G20050.1 | T-complex protein 1 alpha subunit; Molecular chaperone |
|  | eEF-1Bb1 | AT1G30230.2 | Glutathione S-transferase, C-terminal-like;Translation elongation factor EF1B/ribosomal protein S6 |
|  | AT2G18110 | AT2G18110.1 | Translation elongation factor EF1B/ribosomal protein S6 family protein |
|  | ATKRS-1 | AT3G11710.1 | Lysine--tRNA ligase, cytoplasmic |
| Down-Regulted | ORC3 | AT5G16690.1 | Origin Recognition Complex subunit 3. Involved in the initiation of DNA replication |
|  | MCM5 | AT2G07690.1 | Minichromosome maintenance (MCM2/3/5) family protein |
|  | ETG1 | AT2G40550.1 | Mini-chromosome maintenance complex-binding protein |
|  | PLE | AT5G51600.1 | Microtubule associated protein (MAP65/ASE1) family protein |
|  | ATK1 | AT4G21270.1 | Kinesin-like protein KIN-14C |
|  | CYCA3;1 | AT5G43080.1 | Putative cyclin-A3-1; Cyclin A3;1 (CYCA3;1) |
|  | RNR1 | AT2G21790.1 | Ribonucleoside-diphosphate reductase large subunit |
|  | HTA9 | AT1G52740.1 | Probable histone H2A variant 3 |
|  | UBC19 | AT3G20060.1 | Encodes one of two ubiquitin-conjugating enzymes belonging to the E2-C gene family |
|  | AT3G42660 | AT3G42660.1 | Transducin family protein / WD-40 repeat family protein |
|  | CDKB2;2 | AT1G20930.1 | Cyclin-dependent kinase |
|  | CHR1 | AT5G66750.1 | ATP-dependent DNA helicase DDM1 |
|  | AT4G28310 | AT4G28310.1 | Uncharacterized protein At4g28310; Unknown protein |
|  | ORC6 | AT1G26840.1 | Origin of replication complex subunit 6 |
|  | MCM3 | AT5G46280.1 | Minichromosome maintenance (MCM2/3/5) family protein |
|  | MAP65-8 | AT1G27920.1 | Microtubule-associated protein 65-8 (MAP65-8) |
|  | AT1G09200 | AT5G10390.1 | Histone superfamily protein; Core component of nucleosome |
|  | MCM2 | AT1G44900.1 | Minichromosome maintenance (MCM2/3/5) family protein |
|  | BRCA1 | AT4G21070.1 | Encodes AtBRCA1, an ortholog of the human breast cancer susceptibility gene 1 |
|  | CYCA1;1 | AT1G44110.1 | Cyclin A1;1 (CYCA1;1 |
|  | MCM10 | AT2G20980.1 | Minichromosome maintenance 10 |
|  | AT1G14300 | AT1G14300.2 | ARM repeat superfamily protein |
|  | AT5G01230 | AT5G01230.1 | S-adenosyl-L-methionine-dependent methyltransferases superfamily protein |
|  | NRPC2 | AT5G45140.1 | DNA-directed RNA polymerase III subunit 2 |
|  | emb2742 | AT3G12670.1 | CTP synthase family protein |
|  | AT1G48570 | AT1G48570.1 | Zinc finger (Ran-binding) family protein |
|  | AT4G02400 | AT4G02400.1 | U3 ribonucleoprotein (Utp) family protein |
|  | AT4G23540 | AT4G23540.1 | ARM repeat superfamily protein; Its function is described as binding |
|  | AT5G11240 | AT5G11240.1 | Transducin family protein / WD-40 repeat family protein |
|  | TTN5 | AT2G18390.1 | ADP-ribosylation factor family protein |
|  | CMT3 | AT1G69770.1 | DNA (cytosine-5)-methyltransferase CMT3 |
|  | AT1G63100 | AT1G63100.1 | GRAS family transcription factor |
|  | VAM3 | AT5G46860.1 | Syntaxin/t-SNARE family protein |
|  | ENODL14 | AT2G25060.1 | Early nodulin-like protein 14 (ENODL14) |
|  | SYP111 | AT1G08560.1 | Syntaxin-related protein KNOLLE |
|  | AT5G16250 | AT5G16250.1 | Uncharacterized protein T21H19_170 |
|  | CSLD5 | AT1G02730.1 | Cellulose synthase-like protein D5 |
|  | PER64 | AT5G42180.1 | Peroxidase superfamily protein |
|  | RCI3 | AT1G05260.1 | Peroxidase superfamily protein |
|  | AT5G66390 | AT5G66390.1 | Peroxidase superfamily protein |
|  | CAD9 | AT4G39330.1 | Probable cinnamyl alcohol dehydrogenase 9 |
|  | PRX52 | AT5G05340.1 | Peroxidase superfamily protein |
